# Panelist

**DOI:** 10.1002/alz70856_106088

**Published:** 2026-01-08

**Authors:** Sharon J Sha

**Affiliations:** ^1^ Department of Neurology and Neurological Sciences, Stanford University, Stanford, CA, USA

## Abstract

Dr. Sha will be a panelist.

